# Gastric Metastasis of Renal Cell Carcinoma Initially Diagnosed by Esophagogastroduodenoscopy

**DOI:** 10.7759/cureus.79651

**Published:** 2025-02-25

**Authors:** Masaya Iwamuro, Tomohiro Kamio, Shoichiro Hirata, Takehiro Tanaka, Motoyuki Otsuka

**Affiliations:** 1 Department of Gastroenterology and Hepatology, Okayama University Graduate School of Medicine, Dentistry, and Pharmaceutical Sciences, Okayama, JPN; 2 Department of Pathology, Okayama University Hospital, Okayama, JPN

**Keywords:** clear renal cell carcinoma, esophagogastroduodenoscopy (egd), gastric metastasis, metastatic tumor, renal cell carcinoma (rcc)

## Abstract

Here, we report a rare case of renal cell carcinoma (RCC) initially detected as a gastric metastasis. A 58-year-old man with epigastric discomfort underwent esophagogastroduodenoscopy, which revealed a reddish semi-pedunculated lesion with a whitish coating. Biopsy and imaging confirmed clear cell RCC metastasis. Contrast-enhanced computed tomography (CT) revealed a primary renal tumor with pancreatic and lymph node metastases. Despite chemotherapy treatment, the patient died after 10 months. Gastric metastases from RCC, although rare, should be considered in highly vascular gastric lesions with white coatings. Clinicians must be vigilant for metastatic diseases with atypical gastric findings.

## Introduction

Renal cell carcinoma (RCC) is a common malignant kidney neoplasm that accounts for approximately 85% of all renal cancers [1,2]. RCC can metastasize to various organs, including the lungs, bones, liver, and brain [3,4]. However, gastric metastasis from RCC is extremely rare, with an estimated incidence of less than 0.2% among all RCC cases [5]. Due to its rarity, gastric involvement is often not initially considered in the differential diagnosis of gastric lesions, leading to potential delays in diagnosis and treatment.

Diagnosing gastric metastases of RCC presents a significant clinical challenge. In routine clinical practice, gastric lesions are typically evaluated with a focus on primary gastric malignancies, such as adenocarcinoma or lymphoma, or benign conditions like polyps and ulcers. Metastatic tumors, particularly from RCC, can exhibit endoscopic findings that mimic these common gastric pathologies, making early recognition difficult. Furthermore, histopathological confirmation may be challenging, as standard biopsy techniques may fail to obtain sufficient diagnostic material. Given these challenges, clinicians must maintain a high index of suspicion when encountering unusual gastric lesions, particularly those with a highly vascular appearance and a white coating, which may be indicative of RCC metastasis.

Here, we present a rare case of RCC initially detected as a gastric metastasis during esophagogastroduodenoscopy. Endoscopic examination revealed a reddish, semipedunculated lesion with a whitish coating, suggestive of a highly vascular tumor. This case underscores the importance of considering metastatic disease in the differential diagnosis of atypical gastric lesions and highlights the role of endoscopic and immunohistochemical evaluation in establishing a definitive diagnosis. By sharing this case, we aim to raise awareness of this rare presentation and emphasize the need for a systematic approach to diagnosing gastric metastases from RCC.

## Case presentation

A 58-year-old Japanese man presented to a primary care physician with epigastric discomfort and appetite loss. Esophagogastroduodenoscopy revealed a 2 cm elevated lesion in the anterior wall of the upper gastric body. A biopsy was performed; however, the histological findings were inconclusive in diagnosing the pathology of the gastric polyp. After a two-week course of proton pump inhibitors, repeat esophagogastroduodenoscopy with biopsy was performed, revealing no neoplastic cells on histology. The patient was referred to our hospital for further evaluation.

The patient had no relevant medical history. Physical examination revealed no remarkable findings, including those in the abdomen, and no palpable masses or lymphadenopathies. The patient reported consuming approximately 20 g of alcohol and smoked 20 cigarettes daily for 38 years. Blood tests showed mild anemia with a hemoglobin level of 13.0 g/dL, but white blood cell count, platelet count, and biochemical parameters were within normal ranges (Table 1). Tumor markers, including carcinoembryonic antigen and carbohydrate antigen 19-9, were within normal limits.

**Table 1:** Blood test results.

Blood test results (units)	Patient value	Reference range
White blood cells (/μL)	6,150	3,300-8,600
Neutrophil (%)	67.2	40-70
Lymphocyte (%)	24	16.5-49.5
Monocyte (%)	5.7	2-10
Red blood cells (/μL)	4,000,000	4,350,000-5,550,000
Hemoglobin (g/dL)	13	13.7-16.8
Hematocrit (%)	39	40.7-50.1
Platelets (/μL)	307,000	158,000-348,000
Total protein (g/dL)	6.8	6.6-8.1
Albumin (g/dL)	4.4	4.1-5.1
Creatinine (mg/dL)	0.75	0.65-1.07
Sodium (mmol/L)	140	138-145
Potassium (mmol/L)	4.2	3.6-4.8
Total bilirubin (mg/dL)	0.62	0.4-1.5
Direct bilirubin (mg/dL)	0.12	0.08-0.28
Aspartate aminotransferase (U/L)	15	13-30
Alanine aminotransferase (U/L)	12	10-42
γ-Glutamyl transpeptidase (U/L)	28	38-113
Lactate dehydrogenase (U/L)	184	124-222
Alkaline phosphatase (U/L)	246	110-360
C-reactive protein (mg/dL)	0.24	0-0.15
Carcinoembryonic antigen (ng/mL)	3.69	0-5.0
Carbohydrate antigen 19-9 (U/mL)	0.24	0-0.30

**Figure 1:**
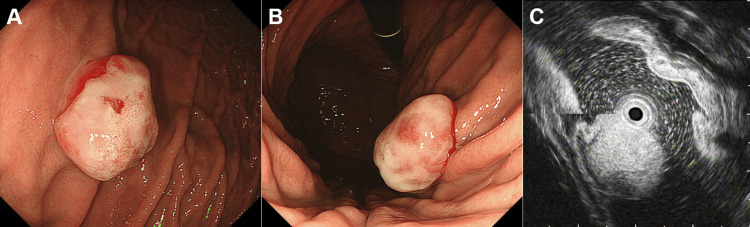
Endoscopic findings of the gastric lesion. Esophagogastroduodenoscopy reveals a reddish, semi-pedunculated elevated lesion on the anterior wall of the upper gastric body, covered with a whitish coating (A, B). Endoscopic ultrasonography demonstrates a hyperechoic mass extending from the mucosal to the submucosal layer (C).

**Figure 2:**
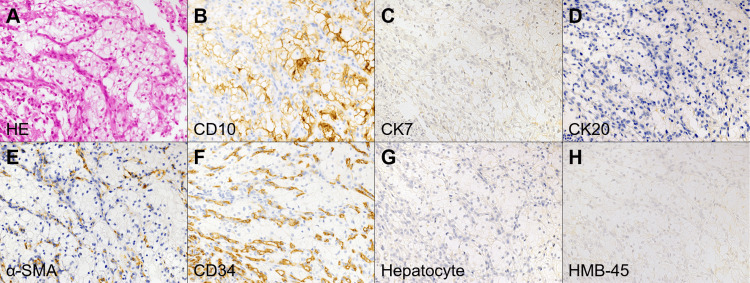
Histopathological and immunohistochemical findings of the gastric lesion. Histological examination shows tumor cells with abundant clear cytoplasm, distinct cell borders, and relatively uniform round nuclei, accompanied by well-developed small blood vessels (A, hematoxylin and eosin stain). Immunohistochemical staining reveals the tumor cells are positive for CD10 (B) and negative for cytokeratin 7 (C), cytokeratin 20 (D), alpha-smooth muscle actin (E), CD34 (F), hepatocyte antigen (G), and HMB-45 (H). The magnification of panels A-H is ×40.

Esophagogastroduodenoscopy performed at our hospital revealed a reddish, semi-pedunculated, elevated lesion covered with a whitish coating (Figure 1A and Figure 1B). Endoscopic ultrasonography revealed a hyperechoic mass extending from the mucosal to the submucosal layers (Figure 1C). A conventional forceps biopsy was performed during esophagogastroduodenoscopy. Biopsy samples included granulation tissue and tumor cells characterized by abundant clear cytoplasm, distinct cell borders, relatively uniform round nuclei (Figure 2A), and well-developed small blood vessels. Immunohistochemical staining showed that the tumor cells were positive for CD10 (Figure 2B) but negative for cytokeratin 7 (Figure 2C) and cytokeratin 20 (Figure 2D). Additionally, the tumor cells show negativity for alpha-smooth muscle actin (Figure 2E), CD34 (Figure 2F), hepatocyte antigen (Figure 2G), and HMB-45 (Figure 2H), ruling out other differential diagnoses such as leiomyoma, gastrointestinal stromal tumors, hemangioma, hepatocellular carcinoma, and melanoma. These findings suggested metastatic clear cell RCC.

**Figure 3:**
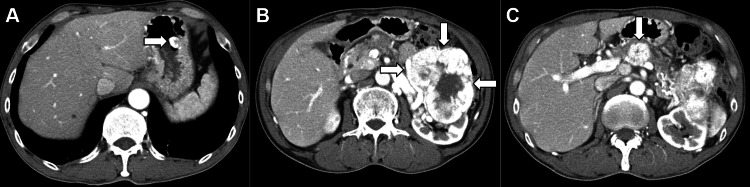
CT findings supporting the diagnosis of metastatic clear cell RCC. Abdominal CT reveals a protruded lesion in the stomach with clear enhancement (A, arrow). A primary tumor in the left kidney with heterogeneous enhancement (B, arrows). Metastatic lesions in the pancreas (C, arrow). CT, computed tomography; RCC, renal cell carcinoma

Contrast-enhanced computed tomography (CT) revealed a protruding lesion in the stomach, showing clear enhancement of the contrast media (Figure 3A, arrow). CT identified a primary tumor in the left kidney (Figure 3B, arrows), along with metastases to the pancreas (Figure 3C, arrow) and lymph nodes. Despite five courses of sunitinib treatment, the disease progressed and was subsequently treated with everolimus. After one course of everolimus, disease progression was observed. The treatment was then switched to temsirolimus, but the disease continued to progress after just one course, and the patient was transitioned to palliative care. The patient died 10 months after diagnosis.

## Discussion

Gastric metastasis from RCC is a rare clinical finding, with a reported incidence of <0.2% of all RCC cases [6]. Although RCC commonly metastasizes to the lungs, bones, and liver [3,4], gastric involvement is rare and often overlooked during diagnosis. Presently, gastric metastasis was the initial presentation that led to the diagnosis of RCC, underscoring the need for clinicians to consider metastatic diseases in the differential diagnosis of gastric lesions.

The average time from RCC diagnosis to the development of gastric metastasis has been reported as approximately seven years (range: 0-23) [7-9]. Interestingly, in cases of solitary gastric metastases, the interval tends to be longer than in cases with multiple metastatic sites [10], highlighting the unique progression pattern of solitary lesions. In this case, the gastric lesion appeared concomitantly with primary RCC and pancreatic and lymph node metastases, serving as a diagnostic clue for RCC, a scenario rarely described in the literature.

Endoscopically, gastric metastases in RCC exhibit diverse morphologies. Commonly observed features include reddish sessile lesions, often accompanied by ulceration or central depression [5,11-13]. Reddish, semi-pedunculated, or pedunculated lesions are frequently noted [14-16]. Presently, white, spherical, semi-pedunculated, or pedunculated lesions are less common but have been documented in several cases [9,17-19]. Additionally, some lesions were described as flat, small spots, or multiple sessile or pedunculated polyps [20], highlighting the variability in presentation. These findings underscore the importance of meticulous endoscopic examinations for identifying metastatic lesions, which can serve as critical diagnostic clues for RCC. Immunohistochemical analysis is indispensable for confirming the diagnosis, as clear cell RCC metastases typically exhibit a clear cell appearance because of the accumulation of glycogen, lipids, and vascular-rich stroma. Neoplastic cell-specific marker profiles include CD10 positivity and cytokeratin 7 negativity.

In this case, two sessions of endoscopic biopsy were performed at the previous institution; however, a definitive diagnosis of the gastric lesion was not achieved. In contrast, our conventional forceps biopsy successfully led to a pathological diagnosis of RCC metastasis to the stomach. The exact differences in biopsy techniques between the previous institution and our approach remain unclear. However, our biopsy samples included not only tumor cells but also granulation tissue, suggesting that inadequate sampling from the appropriate lesion site may have contributed to the initial diagnostic failure. Given this, in cases where the initial biopsy does not yield a diagnosis, repeated biopsies using conventional forceps may be beneficial. Furthermore, considering the possibility of deeper tissue involvement, endoscopic ultrasound-guided fine-needle biopsy may serve as a valuable alternative to improve diagnostic accuracy.

In cases in which a patient presents with a reddish polyp with a white coating, several rare conditions should be considered in the differential diagnosis. These include hypervascular tumors, such as pyogenic granulomas, and metastatic tumors from RCC [9,17-19] because the white coating corresponds to a fibrinous exudate. Pyogenic granuloma is a benign vascular lesion characterized by overgrowth of capillaries and fibrous tissue, typically developing in response to chronic irritation, trauma, or inflammation. Although pyogenic granulomas are more commonly observed on the skin or mucous membranes, such as the oral cavity, gastric occurrences are exceedingly rare. Gastric pyogenic granulomas can appear reddish, solitary, or as multiple polyps, sometimes with a white coating. The stomach can host metastatic tumors in various organs. Although gastric metastases from RCC are rare, they are highly vascular and can present as tumors with a white coating, as described previously.

The prognosis of patients with gastric metastases from RCC is generally unfavorable, with limited long-term survival reported in the literature. Prudhomme et al. reported a median survival of five years for patients with multiple metastatic sites compared to 8.5 years for those with solitary gastric metastases [10]. This highlights the prognostic significance of the metastatic burden in RCC. In this case, the disease progressed rapidly despite systemic chemotherapy, reflecting the aggressive nature of metastatic RCC and the limited efficacy of available treatments in this context. The involvement of multiple metastatic sites, including the pancreas and lymph nodes, likely contributes to a poor prognosis.

## Conclusions

Overall, a reddish protruding lesion with a white coating in the stomach warrants consideration as a tumor with rich vascularity in the differential diagnosis. Gastric metastasis from clear cell RCC, although rare, is an example of a highly vascular tumor. Clinicians should maintain a high level of suspicion for this type of metastatic disease if unusual gastric lesions with a white coating are encountered. This case emphasizes the value of a multidisciplinary approach that integrates endoscopy, histopathology, and imaging to achieve a definitive diagnosis.
